# VNS paired with training enhances recognition memory: mechanistic insights from proteomic analysis of the hippocampal synapse

**DOI:** 10.3389/fnmol.2024.1452327

**Published:** 2024-12-16

**Authors:** Seung H. Jung, Laura K. Olsen, Krysten A. Jones, Raquel J. Moore, Sean W. Harshman, Candice N. Hatcher-Solis

**Affiliations:** ^1^Cognitive Neuroscience, 711th Human Performance Wing, Air Force Research Laboratory, Wright-Patterson AFB, OH, United States; ^2^DCS Infoscitex, Dayton, OH, United States; ^3^Oak Ridge Institute for Science and Education, Oak Ridge, TN, United States; ^4^Integrative Health & Performance Sciences, UES, Inc., Blue Halo, Dayton, OH, United States; ^5^Analytical Chemistry, 711th Human Performance Wing, Air Force Research Laboratory, Wright-Patterson AFB, OH, United States

**Keywords:** vagus nerve stimulation, novel object recognition, recognition memory, proteomics, synaptic signaling, synaptic plasticity

## Abstract

**Introduction:**

Recognition memory, an essential component of cognitive health, can suffer from biological limitations of stress, aging, or neurodegenerative disease. Vagus nerve stimulation (VNS) is a neuromodulation therapy with the potential to improve cognitive function. This study investigated the effectiveness of multiple sessions of VNS to enhance recognition memory in healthy rodents and the underlying cognitive benefits of VNS by proteomic analysis of the synaptosome.

**Methods:**

Rats demonstrated VNS-induced recognition memory improvements using a novel object recognition (NOR) task. Using the LC–MS/MS method, roughly 3,000 proteins in the synaptosome of the hippocampus were analyzed.

**Results:**

Protein–protein interaction (PPI) enrichment analysis found differentially expressed proteins related to synaptic signaling and neurotransmitter pathways. PPI network analysis identified six unique protein clusters, including a cluster of synaptic signaling related pathways. Using ingenuity pathway analysis (IPA), rapamycin-insensitive companion of mTOR was identified as an upstream regulator of synaptosome changes due to VNS-paired training.

**Discussion:**

Based on these results, it is proposed that VNS may mediate cognitive enhancement via increases in glutamatergic signaling and early LTP during the consolidation period, followed by sustained synaptic plasticity via modified post-synaptic receptor expression and dendritic outgrowth. Further investigation is required to determine if VNS is a good candidate to ameliorate cognitive impairment.

## 1 Introduction

Recognition memory is a fundamental component of cognitive health, allowing us to learn and remember new objects, events, or people. This declarative memory process occurs with minimal impedance for most people. However, as input demands begin to exceed our biological limitations, recognition memory starts to decline. Taxing work environments can strain the cognitive capacity of even healthy adults (McKinley et al., 2012). Additionally, recognition memory impairment has been reported among those experiencing chronic stress, aging, and Alzheimer’s disease (Barbeau et al., 2004; Burke et al., 2010; Shamy et al., 2011; Stothart et al., 2021; Wirkner et al., 2019). Vagus nerve stimulation (VNS) is an FDA approved neuromodulation therapy found to enhance multiple elements of cognition, such as arousal, attention, multi-tasking, decision-making, and memory (Giraudier et al., 2020; Martin et al., 2004; McIntire et al., 2021; Sjogren et al., 2002; Sun et al., 2017). However, there is limited knowledge regarding the efficacy of VNS to improve recognition memory and the underlying mechanisms of VNS-induced cognitive enhancement.

The hippocampus (HC) is an essential brain structure for normal recognition memory function (Clark et al., 2000; Cohen and Stackman, 2015; Reed and Squire, 1997). Pre-clinical studies have identified activity-dependent synaptic plasticity within the HC to occur post-training (i.e., the consolidation period) in recognition memory tasks (Clarke et al., 2010; Goulart et al., 2010). Stimulation of the vagus nerve activates cholinergic and noradrenergic pathways, with noradrenergic integration into the hippocampus via the nucleus of the solitary tract and locus coeruleus (Berthoud and Neuhuber, 2000; Cooper et al., 2021; Szabadi, 2013). Application of VNS during the consolidation period has been found to promote synaptic plasticity in rodents, with increases in long-term potentiation (LTP) and brain derived neurotrophic factor (BDNF) in hippocampal sub-regions (Olsen et al., 2022). Although VNS has been shown to promote synaptic plasticity in the HC, a clear understanding of the dynamic signaling events/pathways that occur at the synapse after VNS is required to provide further mechanistic insight into the cognitive benefits of VNS-paired training (Olsen et al., 2022; Sanders et al., 2019; Zuo et al., 2007).

Here, we investigated the effects of multiple sessions of VNS on recognition memory and examined VNS-associated effects on hippocampal protein abundance by proteomics. Proteomics is a powerful form of protein analysis that utilizes technological advances in protein fractionation, mass spectrometry, and bioinformatics. Bioinformatic analysis of the sub-proteome at the synapse (i.e., synaptosome) enables the examination of a comprehensive set of synaptic proteins (including low abundance proteins) (Bai and Witzmann, 2007). Application of this complex technique using liquid chromatography tandem mass spectrometry (LC–MS/MS) produces synaptic peptide fragmentations that are processed to identify differentially expressed proteins (DEPs) and infer protein interactions/signaling pathways. Proteomic analysis of the isolated hippocampal synapse after VNS was conducted to construct a detailed characterization of the synaptic modifications that may mediate VNS-induced cognitive improvements.

## 2 Materials and methods

The views and opinions presented herein are those of the author(s) and do not necessarily represent the views of the Department of Defense (DoD) or its Components. Appearance of, or reference to, any commercial products or services does not constitute DoD endorsement of those products or services. The appearance of external hyperlinks does not constitute DoD endorsement of the linked websites, or the information, products, or services therein.

### 2.1 Animals

This study was reviewed and approved by the Wright-Patterson Air Force Base IACUC in compliance with all federal regulations governing the protection of animals and research. These studies were conducted in a facility accredited AAALAC International, in accordance with the Guide for the Care and Use of Laboratory Animals (NRC, 2011), and were performed in compliance with DODI 3216.01.

Male Sprague–Dawley rats (5–7 weeks old) were obtained from Charles River and group housed in a facility prior to surgery on a 12 h light cycle with *ad libitum* access to water and food. Rats were allowed to acclimate for at least a week before they were exposed to any experimental process. After the acclimation, rats were randomly placed into sham and VNS groups and received a surgery for vagus nerve electrode cuff implantation followed by a recovery period of at least 10 days as described below. After the recovery period, rats received VNS and underwent behavioral tests for 4 days. On the day after the last VNS session (about 24 h post-VNS), rats were euthanized by rapid decapitation for tissue collection and whole hippocampal tissue was collected, immediately frozen, and stored at −80°C.

### 2.2 Vagus nerve electrode cuff implantation and vagus nerve stimulation

While under isoflurane anesthesia (5% induction, 2–3% maintenance), the left cervical vagus nerve was isolated from the carotid artery and unsheathed prior to electrode cuff implantation. Successful surgical implantation of the electrode cuff (90% Pt/10% Ir, 0.0011″ diameter) was confirmed using the cessation of breathing test (Sanchez et al., 2020). After the implantation surgery, rats were singly housed and carefully monitored each day.

At least 10 days post-surgery, rats (10–12 weeks old; 8/group; 16 animals total) underwent VNS and behavioral testing (Supplementary Figure 1). While free moving in an arena (40 cm × 44 cm × 37 cm), rats were administered fifteen 100 μs biphasic pulse trains at 30 Hz, 0.8 mA constant current every 18 s for 30 min using an A365 Isostimulator (WPI). Rats in the sham group received zero mA constant current for 30 min. Pulse train timing was controlled using a CED Micro 1401-3 unit (Cambridge Electronic Design Ltd., UK) paired with Signal 7 software (Cambridge Electronic Design Ltd., UK).

### 2.3 Novel object recognition (NOR) test and analysis

Recognition memory was determined by exposing rats to two similar objects on ‘training day,’ followed 24 h later by a ‘testing day’ exposure to one of the same objects from ‘training day’ and one novel object. Rats habituated to the arenas (60 cm × 60 cm × 38 cm) for 2 min before 3 min exposure to objects on both ‘training day’ and ‘testing day.’ Objects were placed on opposite sides of the arena (47 cm apart). Exploration activities were monitored using Ethovision XT (Noldus Information Technology, version 11.5). Novel Object Preference (NOP) score (Sanders et al., 2019) was calculated using the following equation:

$$NOP=\frac{\left(\mathrm{Nnovel}-\mathrm{Nfamiliar}\right)}{\left(\mathrm{Nnovel}+\mathrm{Nfamiliar}\right)}$$

Rats that exhibited insufficient object exploration behavior (at least 20 s total) were excluded from NOR analyses. NOR data was analyzed for homogeneity of variance and normality. Non-parametric data was analyzed using a Wilcoxon test, while parametric data was analyzed for group differences using a *t*-test or two-way mixed analysis of variance (ANOVA) with Bonferroni post-hoc test using Prism statistical analysis software (GraphPad Software, version 8).

### 2.4 Proteomics sample preparation

Synaptosomes were extracted from dissected rat hippocampal tissue using Syn-PERTM Synaptic Protein Extraction Reagent (ThermoFisher Scientific Inc., MA, USA) as described previously by Jung et al. (2019). A Bradford assay was performed on synaptosome isolations and 100 μg of protein was transferred to a fresh protein lo-bind Eppendorf tube (Enfield, CT, USA). Synaptosomes were centrifuged at 15,000×*g* for 20 min at 4°C, washed once with 200 μL of cold SynPER buffer, and pelleted at 15,000×*g* for 20 min at 4°C. The pellet was resuspended in 100 μL resuspension buffer. Resuspension buffer was prepared as previously described (Hughes et al., 2019). Briefly, samples were reduced with dithiothreitol (DTT, 5 mM final concentration, Sigma-Aldrich, MO, USA) at 60°C for 30 min with shaking. After samples were cooled, cysteines were alkylated with iodoacetamide (15 mM final concentration, Sigma-Aldrich) at ambient temperature for 30 min in the dark. The reaction was quenched by the addition of 5 mM DTT for 15 min and 20 μL of 50 μg/μL washed Sera-Mag beads were added to each sample (GE Health Care, IL, USA). Samples were diluted with 130 μL of 100% ethanol (Sigma-Aldrich) and were mixed in an Eppendorf ThermoMixer at 24°C for 5 min at 1,000 rpm. Protein bound beads were magnetically separated from the solution, the supernatant was removed, and the samples were washed three times with 80% ethanol. Samples were resuspended in 100 mM ammonium bicarbonate (Sigma-Aldrich), 4 μg of sequencing grade Trypsin/Lys-C was added (1:25, Promega Corp., WI, USA), and mixtures were sonicated for 30 s at room temp. Proteins were digested in a ThermoMixer overnight at 37°C and 1,000 rpm. Samples were centrifuged at 18,000×*g* for 1 min at 24°C and beads were magnetically separated from the solution. Supernatants were transferred to new protein lo-bind Eppendorf tubes and vacuum centrifuged to dryness. All samples were resuspended in 25 μL of loading buffer, 2% acetonitrile: 0.03% trifluoracetic acid:H_2_O, and peptide concentration was estimated at 280 nm on a Nanodrop spectrophotometer (Nanodrop, Thermo Fisher Scientific Inc., MA, USA). All samples were diluted to 0.5 μg mL^−1^ with loading buffer and the Nanodrop at 280 nm was repeated for verification.

### 2.5 Bottom-up liquid chromatography mass spectrometry (LC–MS/MS) and data processing

Digested peptides (1 μg) were separated on a Dionex Ultimate 3000 RSLCnano liquid chromatography instrument (Thermo Fisher Scientific Inc., MA, USA). Isocratic preconcentration was performed on a 5 μ, 100 Å, 300 μm × 5 mm C18 PepMap 100 trap column (Thermo Fisher Scientific Inc., MA, USA) at 5 μL/min for 7.5 min with loading buffer. Analytical reversed phase separations were performed at 300 nL/min on a Thermo Fisher Scientific Easy-Spray PepMap 3 μm, 100 Å, 75 μm × 15 cm column. Analytical mobile phases consisted of 0.1% formic acid (aq., A) and 0.1% formic acid in acetonitrile (B, Optima MS Grade, Thermo Fisher Scientific). A 180 min analytical separation was conducted. Briefly, mobile phase compositions were 3% B for 10 min, 30% B for 152 min, 40% B for 157 min followed by a 10 min wash at 90% B and a 10 min equilibration at 3% B. Peptides were ionized with an Thermo Fisher Scientific Easy-Spray source operated at 2.2 kV and detected on an Orbitrap Fusion Lumos mass spectrometer (Thermo Fisher Scientific Inc., MA, USA). MS^1^ scans were acquired at 120,000 resolutions across 375–2,000 m/z. Precursors were selected based on MS (*n* − 1) scans and isolated for data dependent MS^n^ scans in the quadrupole operated with 1.2 m/z isolation window. Fragments were generated by collision induced dissociation (CID) with a 10 ms activation time and a 35% normalized collision energy for +2–+7 precursor charges states in the ion trap utilizing all available parallelizable time over 2 s cycles. Dynamic exclusion for MS^n^ scans was set at a ±10 ppm mass tolerance with exclusion occurring after one time for 15 s.

Tandem mass spectra were searched using the Sequest HT search engine within the Proteome Discoverer Software Suite (v. 2.3, Thermo Fisher Scientific). Briefly, proteomic data was searched against the Uniprot reviewed *Rattus norvegicus* database with the following search parameters: MS^1^ tolerance 10 ppm, an MS^n^ tolerance of 0.5 Da, and three allowed missed tryptic cleavages. Modifications searched included static carbamidomethylation of cysteine, dynamic oxidation of methionine, and dynamic acetylation of the peptide n-terminus. Peptide spectral matches were evaluated utilizing the Percolator algorithm with a maximum Cn of 0.05 and false discovery rate (FDR) targets of 0.05 (relaxed) and 0.01 (strict). Both peptide and protein FDR utilizing *q*-values were held at 0.05 (relaxed) and 0.01 (strict) thresholds. Intensity based precursor ion label free quantitation normalized to total peptide amount was performed with the following settings: unique + razor peptides, retention time alignment <10 min, mass tolerance 10 ppm. The normalized protein abundances were exported from Proteome Discoverer Software Suite for further downstream analysis.

### 2.6 Proteomics data analysis and statistical methods

Normalized abundance data exported from Proteome Discoverer with the minimum FDR value of 0.05 was analyzed with multiple statistical methods. A homoscedastic two-tailed Student’s t-test was used to detect statistical significances of differentially expressed proteins (DEPs) between sham and VNS groups. JMP® Pro (SAS Institute Inc. version 15.2) was used for multivariate analyses, including principal component analysis (PCA) and hierarchical clustering analysis. PCA on the correlation matrix of the normalized abundance values was conducted with the default estimation method. Variable clustering was employed for PCA correlation data across the normalized abundance followed by enrichment analysis for each PCA correlation cluster. Additionally, hierarchical clustering analysis was conducted with the normalized abundance data. Ingenuity pathway analysis (IPA; Qiagen) was used to analyze the proteomics data between sham and VNS groups. Significant DEPs were also analyzed by a method of protein–protein interaction (PPI) network analysis for the organism *Rattus norvegicus* (network edge setting = molecular action; confidence cutoff = 0.7; maximum additional interactors = 0; enrichment cluster coefficient = 0.385; enrichment average degree = 2.46; PPI enrichment *p* = 1.0E−16) and clustered by using the ClusterONE algorithm. STRING database was used for enrichment analysis. Cytoscape (version 3.8) was used to build and analyze the PPI network. Measures of different centralities were calculated to identify critical proteins in the PPI networks by using CytoNCA.

JMP® Pro (SAS Institute Inc. version 15.2) was used for predictive modeling analysis. Predictive analysis was conducted to create possible equations to predict treatment effects or cognitive performance measured by NOR testing. For predictive models for treatments, the top five DEPs based on the absolute value of fold change were chosen. The top nine DEPs based on the highest squared roots of significant Pearson correlation coefficients between Proteomics abundance values and NOR-based cognitive performance were selected to create predictive models. Different predictive analysis methods, including fit generalized analysis, best subset generalized analysis, partition-based predictive analysis, and neural network algorithm-based analysis, were tested to identify the best models. Among different analysis methods, neural network algorithm-based modeling resulted in the best predictive models for treatments (sham vs. VNS) and NOR test-based cognition based on root average squared error values of the models compared. For the neural network algorithm models, two hidden layers were set with TanH, linear, and/or Gaussian activation layers. Samples for training and validation were randomly chosen by the statistical program.

## 3 Results

### 3.1 Multiple sessions of VNS-paired training improve recognition memory in healthy rats

**Figure 1:**
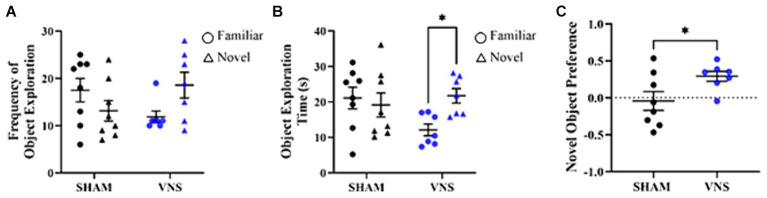
VNS enhances recognition memory in rats. VNS was administered once a day for four consecutive days after behavior testing. **(A)** Stimulated rats showed a trending increase in frequency of object exploration for the novel object as compared to the familiar object. **(B)** Stimulated rats spent more time with the novel object (*p < 0.05 vs. the familiar object). **(C)** The NOP score was enhanced for stimulated rats vs. sham rats (*p < 0.05). Data analyzed using One-way ANOVA and presented as mean ± SEM. Significance values correspond to Bonferroni post-hoc test results.

A rat VNS model was first validated by testing the effect of VNS-paired training on recognition memory through the novel object recognition (NOR) task. The NOR paradigm makes use of the rat’s innate propensity for novelty to quantify recognition memory during exposure to a novel object and a familiar object (Bevins and Besheer, 2006). Rats received a single 30 min session of direct cervical VNS at the same time each day for 4 days in total. VNS was applied 2 days prior to NOR training and testing day to mimic repeated usage of VNS. During the training session, rats were allowed to move freely in an arena and were exposed to two similar objects for 3 min (Supplementary Figure 2). Using a targeted approach of pairing VNS with training (Olsen et al., 2023; Hays et al., 2023), VNS was administered for 30 min after the completion of behavior. The following day, during the testing session, the rats were again placed in the arena but were exposed to one of the same objects from the training day (i.e., familiar) and a new object (i.e., novel). To allow for 4 days of consecutive VNS and tissue collection 24 h after the last session of VNS, another 30 min session of VNS was administered after testing was complete. Four consecutive days of 30 min sessions of VNS was previously found to increase hippocampal synaptic plasticity (Sanders et al., 2019). During the testing session, rats that underwent VNS paired training the previous day demonstrated a trending increase in their interaction with the novel objection versus the familiar object (Figure 1A) and spent more time exploring the novel object (*p* = 0.048, Figure 1B). Controlling for total exploration time, VNS rats had significantly higher Novel Object Preference (NOP) scores compared to the sham group (*p* = 0.045, Figure 1C). In contrast, rats that did not receive VNS did not discriminate between the novel and familiar objects based on frequency of object exploration, object exploration time, or NOP score. As the VNS rodent model was found to produce enhancements in recognition memory, the rodent hippocampal synapse proteome was explored to investigate potential mechanism(s) of action mediating the positive cognitive effects of VNS.

### 3.2 Multiple sessions of VNS modify proteome distribution within the hippocampal synapse

**Figure 2:**
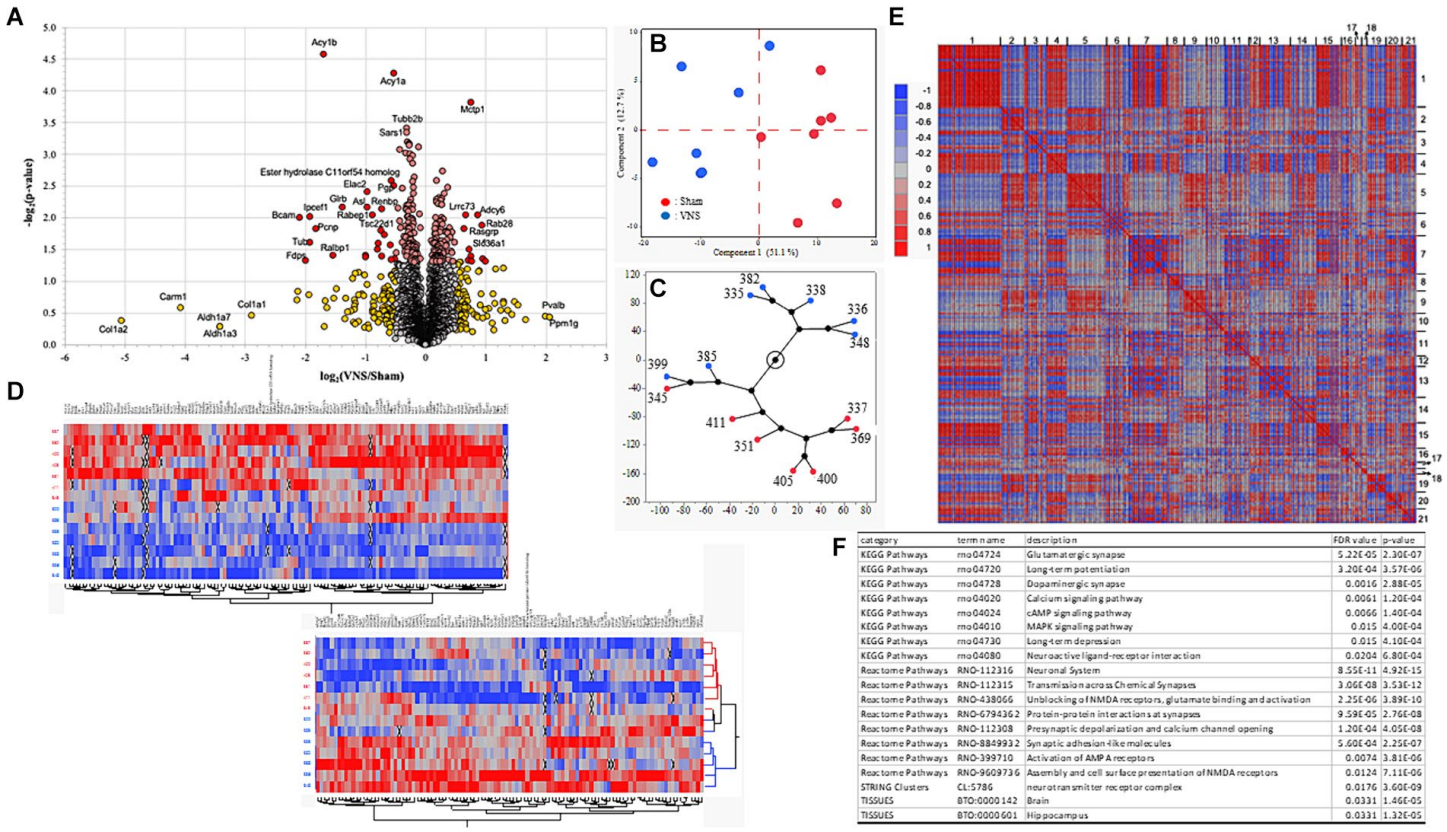
Characteristics of proteomic data. **(A)** Volcano plots for sham and VNS group comparisons. Red and yellow dots represent differentially expressed proteins (DEPs) with statistical significance group differences (*p* < 0.05). **(B)** The distribution of samples from the sham (red) and VNS (blue) groups by PCA. **(C)** Constellation plot from hierarchical clustering analysis shows the distribution of the samples from the sham (red) and VNS (blue) groups. **(D)** Heatmap plot from hierarchical clustering analysis indicate the distribution of each sample across the sham and VNS groups. For clear heatmap plots, see Supplementary Figures 3, 4. **(E)** Heatmap plot of proteomics data clustered by correlation associations. **(F)** Enrichment results from PPI network analysis of cluster-2 identified in panel. **(E)** For all the enrichment results from the clusters, see Supplementary Table 1.

After confirmation of the positive effect of multiple sessions of VNS on improving recognition memory, changes in the proteome at the hippocampal synapse were investigated. Rat hippocampal tissue was collected approximately 24 h after the NOR testing and last session of VNS were completed. Extracted hippocampal synaptic proteins were analyzed using LC–MS/MS and the proteomes of the sham and VNS groups were compared. The abundance level of 3,221 proteins were detected but 2,963 proteins passed criteria (minimum FDR confidence criteria, *q* < 0.05) and were used for further statistical analysis. A significant group difference (*p* < 0.05, two-tailed homoscedastic t-test) was identified for 234 proteins (Figure 2A). Principal component analysis (PCA) and hierarchical clustering analysis showed group separation between the sham and VNS groups (Figures 2B,C). After differentially expressed proteins (DEPs) were analyzed using PCA for hierarchical clustering (Figure 2D) and correlation associations (Figure 2E), PPI enrichment analysis was conducted on cluster-1 and cluster-2. Analysis of cluster-1 identified molecules significantly associated with metabolism-related pathways (Supplementary Table 1). PPI enrichment analysis of cluster-2 found proteins associated with synaptic molecules and neurotransmitter pathways, including glutamatergic synaptic pathway, dopaminergic synaptic pathway, AMPA/NMDA receptor pathways, LTP, LTD, synaptic signaling pathways of calcium, cAMP, and MAPK (Figure 2F and Supplementary Table 1). The abundance levels of the proteins in cluster-2 had significantly higher expression in the VNS group when compared to the sham group (Figure 2D). These findings suggest that VNS modulates the expression of synaptic signaling related proteins within the synaptosome of hippocampal neurons. For deeper proteomic analysis of the synaptosome, ingenuity pathway analysis (IPA) and PPI analysis were performed.

### 3.3 Molecular signaling pathways in the rat hippocampal synapse are modulated by VNS

**Figure 3:**
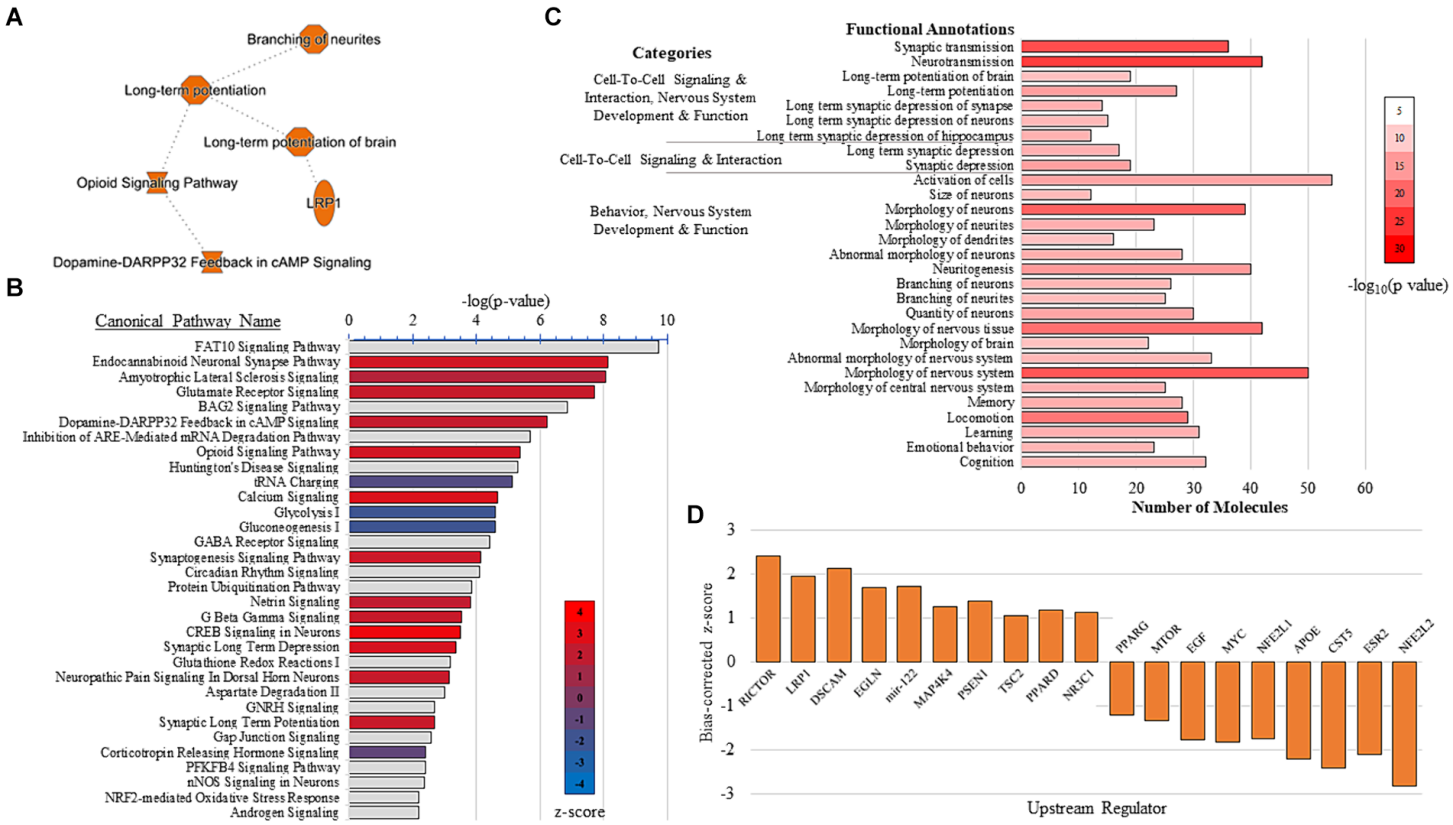
IPA results. **(A)** IPA graphical summary shows VNS enhanced LTP and neurite branching. **(B)** IPA canonical pathway analysis of the top 30 pathways detected from the comparison between the sham and VNS groups. VNS increased pathways in red and decreased pathways in blue. Gray bars have no *z*-score. For all results from the IPA canonical pathway analysis, see Supplementary Table 2. **(C)** Functional annotations detected from the combination of five IPA-generated networks associated with the nervous system. For all results from the IPA network analysis, see Supplementary Tables 3, 4. **(D)** Top activated and inhibited upstream regulators detected from the IPA upstream analysis. For all the results from the upstream regulator analysis, see Supplementary Table 5.

IPA and PPI analysis were conducted to explore the effect of VNS-paired training on synaptosome pathways, networks, upstream regulators, and predictable protein interactions. Summary results from the IPA show VNS enhanced LTP and branching of neurites (Figure 3A). The IPA canonical pathway analysis resulted in 205 significant pathways (*p* < 0.05, Supplementary Table 2) and the top 30 pathways effected by VNS are depicted in Figure 3B. Multiple sessions of VNS were found to increase glutamate receptor signaling, synaptogenesis signaling, and calcium signaling. Conversely, VNS decreased metabolism-associated signaling, such as glycolysis and gluconeogenesis.

The IPA generated 14 networks from significant DEPs (Supplementary Table 3). Five IPA networks (Network IDs 1, 2, 5, 6, and 10) were combined as their top individual functions were all associated with nervous system development and function. Many of the molecules in the combined network were significantly associated with functions that are known to influence cognitive performance, including LTP, memory, learning, cognition, and branching of neurites/neurons (Figure 3C and Supplementary Table 4). IPA was also used to identify upstream regulators that may mediate the synaptic plasticity changes observed after multiple sessions of VNS (Figure 3D). The IPA upstream regulator analysis detected 198 upstream regulators (Supplementary Table 5). Rapamycin-insensitive companion of mTOR (RICTOR) was the top activated upstream regulator and interestingly, seven molecules that are targeted by RICTOR were also detected. Overall, the top activated upstream regulators were LRP1, DSCAM, EGLN, mir-122, MAP4K4, PSEN1, TSC2, PPARD, and NR3C1. The top inhibited upstream regulators were NFE2L2, ESR2, CST5, APOE, NFE2L1, MYC, EGF, MTOR, and PPARG.

**Figure 4:**
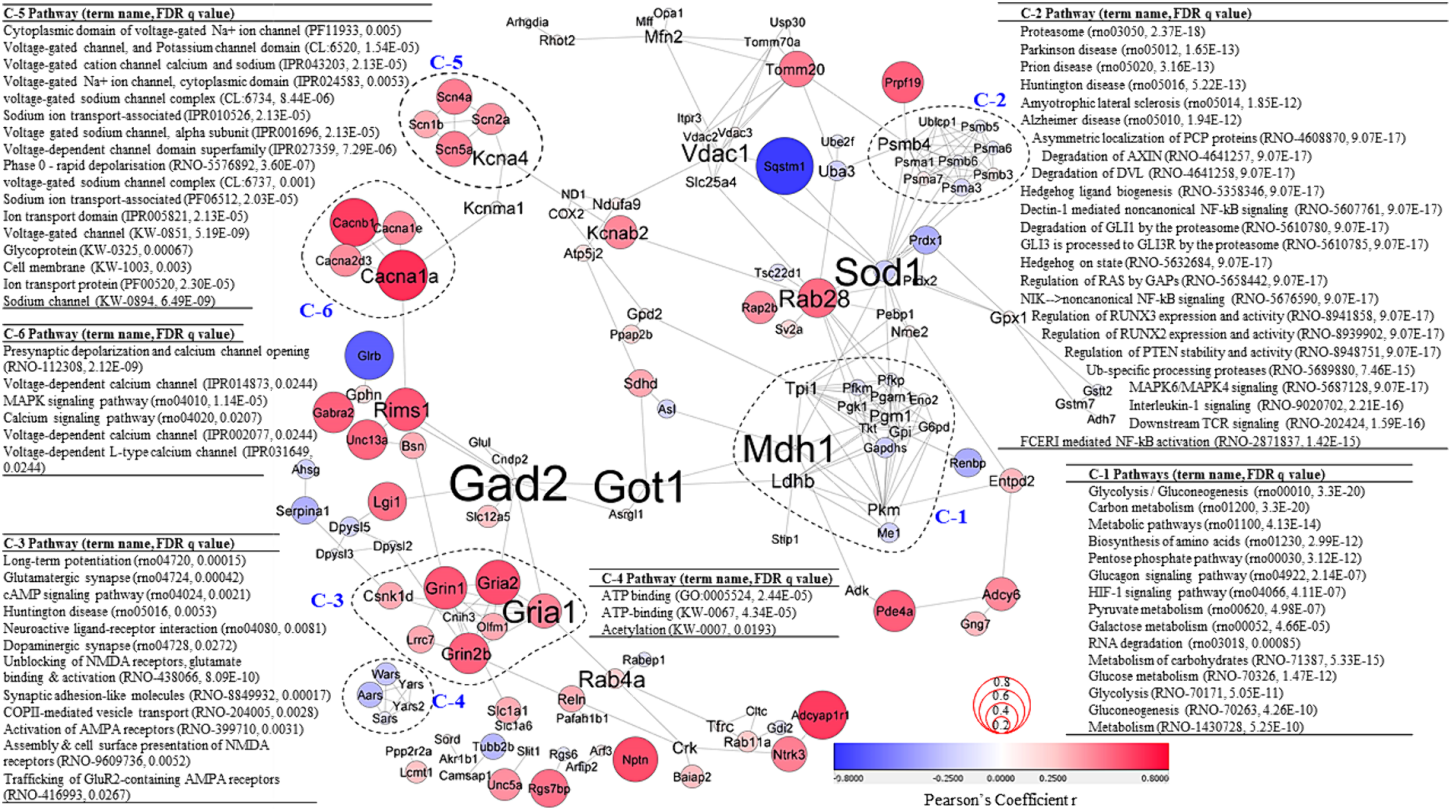
PPI network analysis. PPI network created using significant DEPs (*p* < 0.05; confidence cutoff 0.7; maximum additional interactors = 0). Subgraphs were clustered based on the ClusterOne algorithm. Node color and size represent Pearson’s coefficient *r* values calculated between protein abundance values and rat NOR NOP scores. Font size of protein names represents measures of betweenness centrality. Significant pathways for each cluster are highlighted. For all the results from the PPI network analysis, see Supplementary Tables 6–8.

To better understand the effects of VNS on molecular interactions, significant DEPs were used to create PPI networks (Figure 4). Correlative coefficient r values were calculated for abundance data of all proteomics data and 136 proteins were identified with abundance values significantly associated with NOP score (*p* ≤ 0.05, Supplementary Table 6). Correlation coefficient values on the PPI network are denoted by node color and size in Figure 4 to describe the relationship between the clusters. To further investigate the PPI network, a clustering analysis and PPI enrichment analysis were completed. As the graph clustering algorithm, ClusterONE, is known to be suitable for detecting protein complexes in PPI networks with associated confidence, this algorithm was used for the PPI network analysis (Nepusz et al., 2012). A total of 25 clusters were generated (Supplementary Table 7). Each statistically significant individual cluster (*p* < 0.01) was analyzed again using PPI enrichment analysis and more than 600 enrichment terms for all six clusters (mean = 104 terms & median = 87 terms) were discovered (Supplementary Table 8).

**Figure 5:**
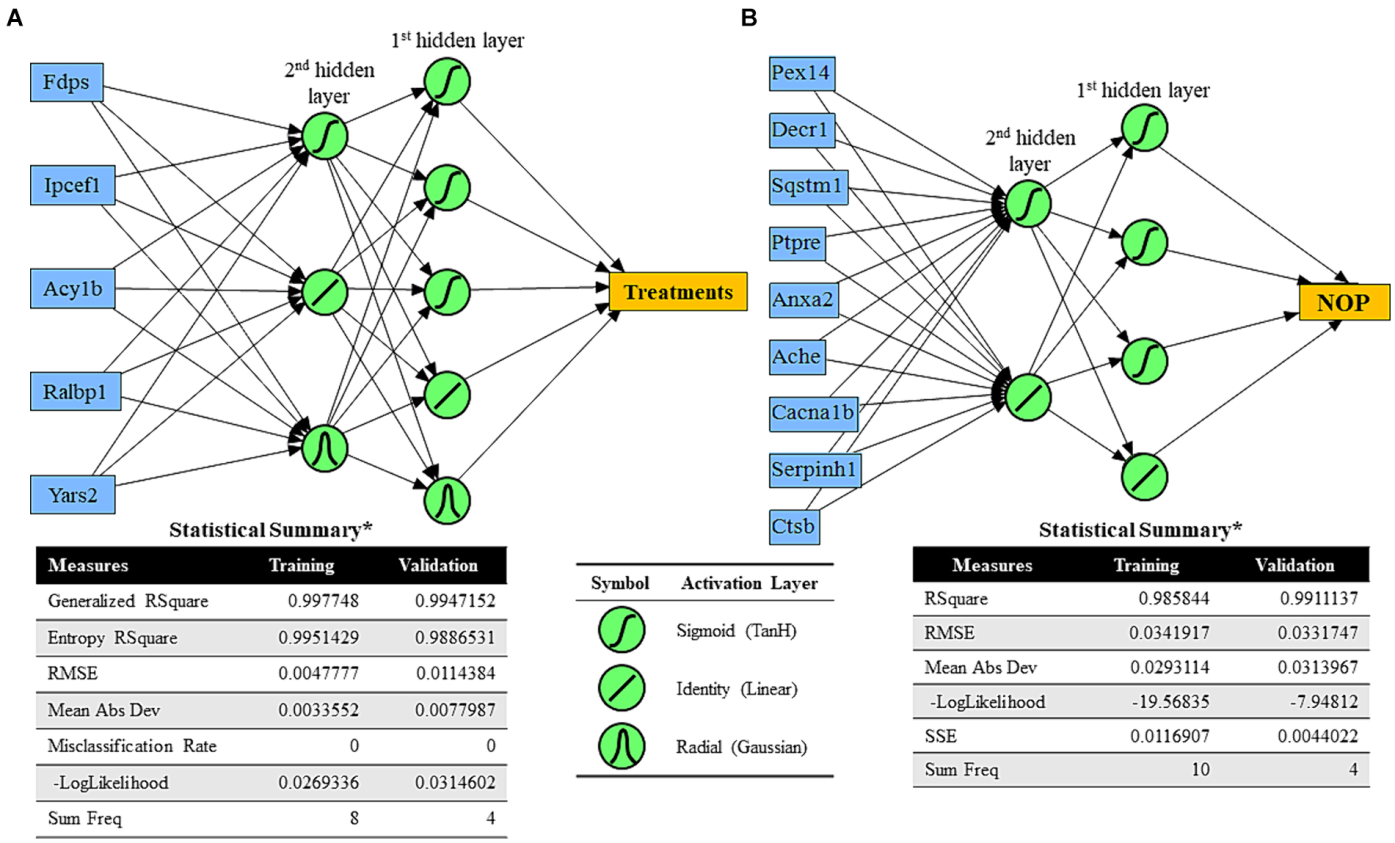
Predictive analysis results of neural network algorithms. **(A)** Neural network algorithm model for treatments (sham vs. multiple VNS) using five significant DEPs based on the absolute value of fold change between the sham and VNS groups (blue boxes) and two hidden layers with TanH, linear, and Gaussian activation functions. Statistical results of training and validation were determined. **(B)** Neural network algorithm model for cognitive performance of rats (NOP) using nine significant DEPs based on the highest squared roots of significant Pearson correlation coefficients between Proteomics abundance values and NOR-based cognitive performance (blue boxes) and two hidden layers with TanH and linear activation functions. Statistical results of training and validation were determined.

Cluster-1 (C-1) was associated with metabolism pathways and the molecules in C-1 were often negatively correlated with NOP score. The proteins in cluster-2 (C-2) were also mostly negatively correlated with NOP score and were significantly associated with Alzheimer disease (rno05010, FDR *q* = 1.94E−12), MAPK6/MAPK4 signaling (rno5687128, FDR *q* = 9.07E−17), and NF-kB signaling (rno5676590, FDR *q* = 9.07E−17; rno5607761, FDR *q* = 9.07E−17), and NF-kB activation (rno2871837, FDR *q* = 1.42E−15). Cluster-3 (C-3) was positively correlated with NOP score. The PPI enrichment analysis for C-3 identified significant pathways and functions associated with cognition, including long-term potentiation (rno04720, FDR *q* = 0.00015), glutamatergic synapse (rno04724, FDR *q* = 0.00042), cAMP signaling pathway (rno04024, FDR *q* = 0.0021), neuroactive ligand-receptor interaction (rno04080, FDR *q* = 0.0081), dopaminergic synapse (rno04728, FDR *q* = 0.0272), unblocking of NMDA receptors, glutamate binding & activation (rno438066, FDR *q* = 8.09E−10), activation of AMPA receptors (rno399710, FDR *q* = 0.0031), assembly & cell surface presentation of NMDA receptors (rno9609736, FDR *q* = 0.0052), and trafficking of GluR2-containing AMPA receptors (rno416993, FDR *q* = 0.0267). All protein abundance values of Cluster-4 (C-4) were negatively associated with NOP score. C-4 was significantly associated with ATP binding (GO:0005524, FDR *q* = 2.44E−05; KW-0067, FDR *q* = 4.34E−05) and acetylation (KW-0007, FDR *q* = 0.0193). Cluster-5 and Cluster-6 (C-5 and C-6) are significantly associated with channel/ion transport-associated functions and pathways. Positive correlations with NOP score were detected for protein abundance levels of C-5 and C-6. Measures of betweenness centrality were calculated to detect which proteins play important roles in the network (Figure 5, depicted by font size of protein names). The measures of betweenness centrality identified Gad2, Got1, Mdh1 (in C-1), Sod1, and Gria1 (in C-3) as highly important in the network.

### 3.4 Neural network algorithm can predict treatment group and recognition memory performance

Machine Learning (ML) algorithms can be beneficial for predicting human behavior, including modified behavior from neuromodulation treatment. An early step toward understanding the relationship between protein changes in the synaptosome and predicting cognitive behavior, protein abundance data were applied to create models which predicted treatment group and recognition memory performance as measured by NOP score. To generate a predictive ML model for treatment group (sham or VNS), five significant DEPs with the largest fold changes were selected (i.e., Fdps, Ipcef1, Acy1b, Ralbp1, Yars2). Using randomly chosen training and validation samples, a predictive ML model (using two hidden layers with TanH, linear, and Gaussian activation functions) was generated with generalized *r*^2^ > 0.99 (Figure 5A). The ML model for treatment group was a predictive model of categorized data, so the ML model calculated percent likelihood of being sham or VNS and chose the prediction based on the highest percent value. To generate a predictive ML model for cognitive performance based on NOP score (regardless of treatment group), nine proteins with the highest correlation values to NOP score were selected (i.e., Pex14, Decr1, Sqstm1, Ptpre, Anxa2, Ache, Cacna1b, Serpinh1, Ctsb). With randomly chosen training and validation samples, a predictive ML model for NOP score was generated (using two hidden layers with TanH and linear activation functions) with *r*^2^ = 0.986 (training) and *r*^2^ = 0.991 (validation).

## 4 Discussion

In this study, we examined whether multiple sessions of VNS could improve performance in a novel object recognition task and the associated protein abundance changes in the HC. Multiple sessions of VNS enhanced recognition memory in healthy male rats. Mechanistic investigation using proteomic analysis suggests synaptic plasticity in the HC is correlated with improved cognitive performance after VNS paired training. Our results indicate VNS induces changes in hippocampal synaptosomes and identified pathways related to cognition, memory, and learning were enhanced.

While recognition and working memory have been shown to be affected by VNS in clinical studies, the improvements to memory have been inconsistent (Olsen et al., 2023). Understanding the mechanisms of action for VNS-induced memory enhancements could facilitate optimization efforts to maximize the effects of VNS. We therefore utilized a pre-clinical rodent VNS model to investigate the mechanism of VNS-induced cognitive enhancement. Multiple sessions of VNS (including a VNS-paired training session) effectively increased recognition memory in rats as measured by the NOR task. Previous rodent studies have also found mild direct cervical VNS to improve performance in the NOR task (Olsen et al., 2022; Sanders et al., 2019). Based on the literature and this study’s behavioral results, it is suggested that the rodent VNS model is suitable for reliably measuring VNS effects on recognition memory and could be utilized to provide mechanistic insights for the improved cognitive performance.

Proteomics analysis of the hippocampal synapse was conducted to understand the associated mechanisms for VNS-induced cognitive enhancement. There were significant differences in protein abundances between the VNS and sham group. Clustering analysis indicated separation in distribution between the groups. Protein abundance and PPI network analyses found VNS promotes synaptic signaling, including neurotransmitter (glutamatergic and dopaminergic) and neuronal excitation pathways. Previous studies have found LTP to be increased after VNS (Olsen et al., 2022; Van Lysebettens et al., 2020; Zuo et al., 2007). The findings from the current study suggest VNS-mediated changes in LTP may be facilitated by glutamatergic signaling pathways. Interestingly, noradrenaline has been found to modulate glutamatergic signaling and these pathways have been suggested to synergistically modify neuronal excitation and memory consolidation (Egli et al., 2005; Mather et al., 2016). Our proteomic analysis identified modifications to AMPA and NMDA receptors, including the unblocking of NMDA receptors and trafficking of AMPA receptors, which could lead to enhanced depolarization of neurons in the hippocampus. As such, AMPA/NMDA receptor modifications may promote VNS-induced increases in LTP and these synaptic plasticity events in the HC could play a role in VNS-enhanced recognition memory.

As shown in our results, synaptic plasticity in the HC was identified to be significantly associated with VNS-induced cognitive enhancement. In addition to synaptic signaling modification, IPA of synaptosome proteins identified upstream regulators that were modulated after VNS. The top activated regulator was an mTOR2 specific regulator, RICTOR (Sarbassov et al., 2004). The RICTOR-mTOR2 complex is known to modulate the phosphorylation of actin cytoskeleton (Sarbassov et al., 2004). Previous studies investigating the function of RICTOR in the brain found deletion or knock down of RICTOR to result in reduced neuronal size, simplified dendritic arbor morphology, and impaired LTP (Huang et al., 2013; Thomanetz et al., 2013; Urbanska et al., 2012). Specific to the HC, RICTOR is suggested to mediate actin polymerization and dendritic outgrowth of hippocampal neurons, with RICTOR deletion leading to late phase LTP and long-term memory impairment (Huang et al., 2013; Urbanska et al., 2012). Supporting the potential for VNS to promote structural remodeling, a previous pre-clinical study found VNS to increase dendritic complexity in immature hippocampal neurons (Biggio et al., 2009). These findings suggest that VNS may produce sustained synaptic plasticity in the HC due to dendritic arborization. Highlighting the relevance of synaptic plasticity and structural remodeling to cognitive enhancement, the ML model to predict recognition memory performance included DEPs with synaptic structure modifying functions, including membrane organization, acting remodeling, and membrane anchors for microtubules (Bharti et al., 2011; Grindheim et al., 2017). Although the predictive ML models are limited due to the relatively small sample sizes in this study, they provide the framework for future models that consider treatment and memory performance to potentially predict the effectiveness of a VNS treatment to produce an enhanced memory outcome.

Altogether, this study establishes the effectiveness of VNS paired training to enhance recognition memory and suggests that synaptic plasticity events mediate the cognitive benefits of VNS. Based on proteomic investigation into the hippocampal synapse, it is proposed that VNS may mediate cognitive enhancement via increases in glutamatergic signaling and early LTP, followed by sustained synaptic plasticity via modified post-synaptic receptor expression and dendritic outgrowth. These mechanistic insights allow for more targeted approaches to be developed for the utilization of VNS to improve cognition.

## Data Availability

The raw data presented in this study can be found below, further enquiries should be directed to the corresponding author(s): https://storage.googleapis.com/afrl-il2-backup-gcs-padata-xtb5/10.3389/fnmol.2024.1452327/AFRL-2024-5866_Synapto_Data-20241122T133027Z-001.zip.
