# Supplementary material for: VNS paired with training enhances recognition memory: mechanistic insights from proteomic analysis of the hippocampal synapse
Source: Front Mol Neurosci. 2024 Dec 16;17:1452327. doi: 10.3389/fnmol.2024.1452327 (PMC11685747; doi:10.3389/fnmol.2024.1452327)
Supplement: Supplementary file 1 [file Data_Sheet_1.docx]

***Supplementary Material***

**VNS paired with training enhances recognition memory: Mechanistic insights from proteomic analysis of the hippocampal synapse**

**S.H. Jung✝^1,2^, L.K. Olsen✝^1,3*^, K.A. Jones^1,4^, R.J. Moore^1,2^, S.W. Harshman^5^, and C.N. Hatcher-Solis^1^**

✝ These authors contributed equally to this work and share first authorship

^1^Cognitive Neuroscience, 711^th^ Human Performance Wing, Air Force Research Laboratory, Wright-Patterson AFB, OH, United States

^2^DCS Infoscitex, Dayton, OH, United States

^3^Oak Ridge Institute for Science and Education, Oak Ridge, TN, United States

^4^Integrative Health & Performance Sciences, UES, Inc., Blue Halo, Dayton, OH, United States

^5^Analytical Chemistry, 711^th^ Human Performance Wing, Air Force Research Laboratory, Wright-Patterson AFB, OH, United States

*** Correspondence:**Laura K. Olsen
laura.olsen.1.ctr@us.af.mil

**Table of Contents**

Supplemental Figure 1 S2

Supplemental Figure 2 S3

Supplemental Figure 3 S4

Supplemental Figure 4 S5

Supplemental Tables 1-8 Provided as Excel Documents

**
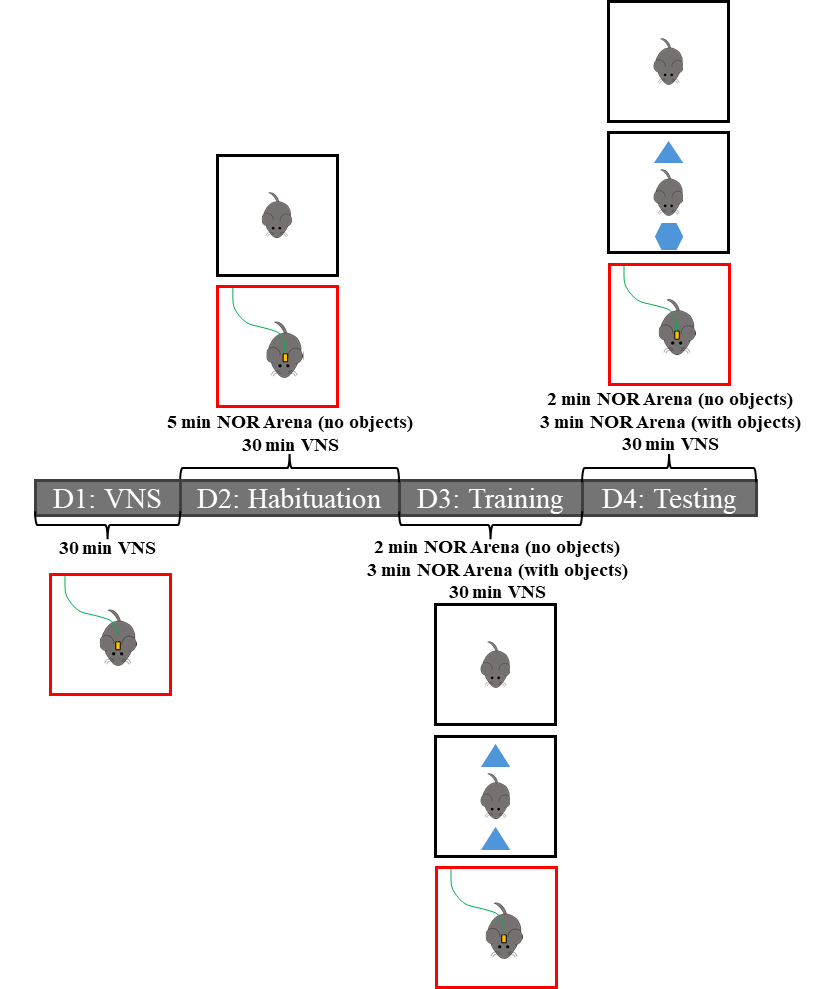
**

**Supplementary Figure 1.** Novel Object Recognition paradigm and Vagus Nerve Stimulation timeline. D1 = Day 1, D2 = Day 2, D3 = Day 3, D4, = Day 4, VNS = Vagus Nerve Stimulation


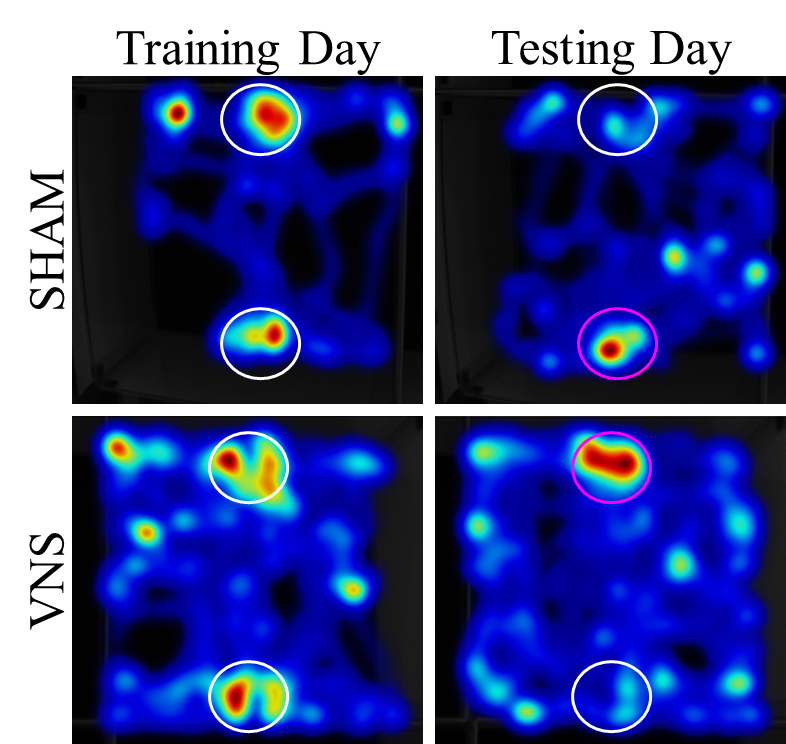


**Supplementary Figure 2.** Representative Novel Object Recognition occupancy heatmap of nose-point cumulative exploration time in arena during object exploration on training and testing day. Arenas are sized 60 cm x 60 cm x 38 cm and objects are placed on opposite sides of the arena 47 cm apart. White circle = familiar/same object, Pink circle = novel object

**Supplementary Figure 3.** Heatmap plot from hierarchical clustering analysis indicate the distribution of each sample across the sham and VNS groups.

**Supplementary Figure 4.** Heatmap plot from hierarchical clustering analysis indicate the distribution of each sample across the sham and VNS groups.
